# Sport practice, physical structure, and body image among university students

**DOI:** 10.1186/s40337-017-0163-1

**Published:** 2017-10-17

**Authors:** Stefania Toselli, Federico Spiga

**Affiliations:** Universita degli Studi di Bologna, Bologna, Italy

**Keywords:** Body image, Body dissatisfaction, Sport practice, Weight status

## Abstract

**Background:**

The aim of this study was to estimate body image perception in undergraduate students, and to investigate its associations with weight status, abdominal obesity, muscularity, gender and sport.

**Methods:**

The sample consisted of 231 Italian students (174 males and 57 females); anthropometric measurements, taken by trained technicians, were: height, weight, arm-circumference, waist and hip circumferences. BMI, WHR and Δ arm-circumference were calculated. Body image was assessed using body silhouette charts. Information about sport (currently practiced sport, starting age, and weekly hours of sport) was acquired with questionnaires.

**Results:**

Females perceived themselves as slightly overweight, while males identified themselves as normal weight. Females had a tendency to desire to be thinner in all weight status categories; in males, normal weight subjects had a tendency to desire to be larger, while overweight wished to be thinner. Sport practice was significantly higher in males. Individuals who were overweight and did less sport were significantly more likely to have higher body dissatisfaction.

**Conclusions:**

The present study highlights a positive relationship between sport practice, corpulence and body image perception.

## Plain English summary

The aim of this study is to research associations between body image, obesity, muscularity, gender and sport among university students. The sample consisted of 231 subjects; anthropometric measurements, body image and information about sport were acquired by trained technicians. Females perceive themselves as slightly overweight and wished to be thinner, normal weight males wished to be larger, while people who were overweight wished to be thinner. Sport practice was significantly higher in males. People who were overweight and did less sport were significantly more likely to have higher body dissatisfaction.

The present study highlighted the importance of sport in improving self-consciousness, well-being, and health. Encouraging sport practice may improve self-determination, self-acceptance, and pursuing these positive effects may motivate individuals to initiate and maintain regular exercise routines.

## Background

Body self-esteem is multidimensional and consists of self-evaluation, as well as others’ perception of body weight and general appearance [1]. Body self-esteem is strongly related to overall self-esteem [1, 2], which is not surprising because the media in Western societies presents images of the ideal body and wields pressure for women to have a very thin body and men to have a big and muscular body [3–5].

Self-confidence is strongly influenced by body image in youth populations [6]. While this aspect has been widely studied in women, it is also an important predictor of psychological well-being in young men [7]. Furthermore, gender is often identified with characteristics and traits considered optimal for males and females that constitute masculinity and femininity, respectively [3, 8]. Previous research suggests that gender influences the perception of healthy lifestyles and health-related decisions [9–11].

It is moreover important to consider that the media’s representation of males and females is changing. In particular, “drive for muscularity”, the extent to which an individual wish to increase his muscularity, is a growing issue, and it brings its own set of associated health problems, such as increased level of eating pathology [12].

The media displays women’s bodies as not only thin, but also athletic and toned, and the drive for muscularity in Western women could be due to the emphasis the society gives to physical exercise as part of an ideal lifestyle [13]. So, an intervention aimed to treating and preventing body image concerns may well include a focus on not just thin ideals but also on thinness linked with toned muscles, to critically evaluate unrealistic ideals that the individuals are striving to attain [14]. Contemporary Western culture has also become increasingly focused on male bodies and male body image has changed along with cultural and societal values. Young men feel a social expectation to be more muscular, and this may be associated with social power and self-confidence [15–17]. It is not surprising that men are joining their female peers in the experience of body dissatisfaction [18]. Thus, the body prototypes for both men and women have changed suggesting goals not always easy to achieve [18, 19] and this has had a negative impact on self-esteem, body dissatisfaction and drive for muscularity in both men and women [13, 14]. In addition to gender differences, physical activity plays an important role in body self-esteem in Western society and culture, and a toned body is desirable for the presentation of oneself to others [20–24]. Positive body image perception leads to a sense of efficacy, self-determination, personal acceptance and self-acceptance, which are all closely linked to the development of self-esteem.

Misperception of weight, defined as the difference between the individual’s perceived weight status and his/her actual weight status, can influence health behaviors [25].

Studies show that overweight misperception varies by gender, as females perceive themselves as overweight more frequently than males of the same body mass index (BMI) do [9, 26]. Misperception of overweight/obesity among adolescents of normal weight can have negative consequences as body dissatisfaction, may influence diet and consequently trigger the development of eating disorders [26]. In addition, adolescents who undergo dietary restriction and unhealthy or “do-it-yourself” weight control are at an increased risk of weight gain over time [27]. In recent years there has been an increasing prevalence of eating disorders in young men, probably also because of the negative influences of the mass media mentioned above. It was found that 10%–20% of the cases of anorexia nervosa and bulimia nervosa, and up to 40% of cases of binge eating disorders, occur in men [28]. The role that weight status has on body dissatisfaction in men requires hence further investigation.

With regard to the aims of the present study it is important to note that using BMI alone, the most used indicator to define weight status, has numerous limitations. It does not consider differences in fat mass and fat free mass/muscle mass, leading to incorrectly classifications [28]; in addition, it does not consider body fat distribution that has important health risks implications. Furthermore, body fat distribution is a mediating factor for body size estimation accuracy. In their study on obese women, Rhodes & O’Neil [29] reported that those with lower body fat distribution tended to underestimate their body size and professed to feel thinner than they actually are, while those with upper and mixed-type body fat distributions tended to overestimate their body size and feel heavier than they actually are. Since their results concerned obese women, the reliability of these findings also on non-obese subjects of both sexes deserves to be evaluated, considering other measurements, such as circumferences, muscularity and fat distribution.

Thus, our study goals were to evaluate body image perception, main health-related anthropometric characteristics (considering in addition to weight status, also muscularity and fat distribution) and sport practice in second-year undergraduate male and female students attending university, as well as to explore the impact of gender on these variables and to study the associations between them. In the literature female students generally present a lower BMI and a higher dissatisfaction than males and engage in less physical exercise. However, given the changes in body image perception to which people, particularly youth, are subject to, we wanted to investigate associations between weight status and body image perception in both sexes.

Furthermore, since using BMI alone to define weight status has limitations, and since muscularity and fat distribution are informative indicators another aim was to examine their role as possible mediating factors in body image perception and dissatisfaction.

## Methods

A cross-sectional study of 231 university students aged 21.4 ± 2.0 years was conducted during the 2013–2014 academic year. The subjects (174 males and 57 females) were attending the School of Pharmacy, Biotechnology and Sports Sciences at the University of Bologna (Italy) and assessed during the laboratory lessons for the Anthropometry and Ergonomics course in the second year of the degree program.

The study was approved by the Bioethics Committee of the University of Bologna; all participants gave their consent for the study and had the right not to participate and/or withdraw from the study at any time.

A self-administered questionnaire obtained demographic (gender, age) and sport participation information (sport practice - considered as club sports, namely planned sports activities led by staff - and sport hours per week). Anthropometric measurements were taken in private by trained technicians in the University laboratory using standardized procedures [30, 31]. Height was recorded to the nearest 0.1 cm with a stadiometer, and weight was measured to the nearest 0.1 kg with a high-precision mechanical scale. Body mass index (BMI) was calculated as weight (kg) divided by height squared (m2). Students were classified as underweight, normal-weight, overweight, and obese based on the World Health Organization (WHO) cutoffs [32]. As there was only one obese subject (one male), he was included in the overweight group for further data analysis. For measurement of body circumferences, relaxed arm circumference was measured at the midpoint between the acromion and the olecranon using a non-stretch tape.

Contracted arm circumference was assessed by the same method but with the arm flexed at 90° and the biceps muscle fully contracted. Delta arm circumference (Δ arm circumference) was calculated by subtracting relaxed arm circumference from contracted arm circumference. Waist circumference was measured at the midpoint between the lower border of the rib cage and the iliac crest using a flexible inch tape. Hip circumference was measured at the widest portion of the buttocks. Circumferences were measured over the naked skin and recorded to the nearest 0.1 cm. Waist-Hip Ratio (WHR) was calculated as waist circumference divided by hip circumference.

Body image perception was assessed using body silhouette charts [33]. Subjects were shown nine male or female silhouettes (marked F.1, F.2, F.3, F.4, F.5, F.6, F.7, F.8, and F.9), ordered in morphology from emaciation to obesity. Subjects were asked to select the silhouette that they believed was most similar to their own (‘actual’ figure), as well as the silhouette that they most desired (‘ideal’ figure). Using the classification recommended by Sánchez-Villegas et al. 2001, the silhouette series were divided into four categories (underweight, normal weight, overweight, and obese): Figs. 1, 2 and 3 correspond to “underweight” status, Figs. 4 and 5 correspond to “normal weight” status, Figs. 6 and 7 correspond to “overweight” status, and Figs. 8 and 9 correspond to “obese” status. A body dissatisfaction score (BDS) was calculated by subtracting the “ideal figure” from the “actual figure” score. A positive BDS score indicated the actual figure was larger than the ideal figure and a negative score indicated the actual figure was thinner than the ideal figure A BDS score of 0 indicated no discrepancy (same figure chosen as actual and ideal) [34].

Physical activity frequency of each subject was determined by the hours of sport training during a typical week as declared by the subject.

Data analysis was performed using Statistica for Windows, version 7.1 (StatSoft Italia srl, Vigonza, Padua, Italy). Data were expressed as mean and standard deviation. Comparisons between the genders and between subjects who do or do not engage in sport were performed using Student’s t-test and non-parametric Mann-Whitney U tests for normal and non-normally distributed variables, respectively.

Comparisons among weight status and sport practice frequencies were performed using chi-square (and Fisher’s exact tests when appropriate). Body image perception in relation to weight status category was analyzed using analysis of variance (ANOVA).

Pearson’s correlation analysis were computed between actual self, ideal self, and BDS and anthropometric characters (BMI, muscularity and fat distribution). Multiple linear regression models were performed. Actual self, ideal self, and BDS were considered to be dependent variables. Gender, BMI, Δ arm circumference, WHR, sport practice, sport hours per week were considered to be independent variables, using a three-step model.

In the first model we included gender. In the second model, we also included the anthropometric characteristics (BMI, Δ arm circumference, WHR) of the participants. In the third model sport practice and sport hours per week were finally included. Significance was set at *p < 0.05*.

## Results

Mean values for anthropometric characteristics and body image perception according to gender are reported in Table 1.

**Table 1:** Sample characteristics, weight status and sport practice by gender

	Males	Females	*p* value	*t* value	t (df)	chi-square
Sample characteristics*	Mean (SD)	Mean (SD)				
Height (cm)	177.6 (7.0)	165.3 (6.5)	<.001	11.77	229	
Weight (kg)	73.6 (9.5)	59.2 (6.6)	<.001	10.62	229	
Body Mass Index (BMI)	23.3 (2.4)	21.6 (2.1)	<.001	4.60	229	
Relaxed Arm Circumference (cm)	29.1 (2.9)	26.2 (2.6)	<.001	6.87	229	
Contracted Arm Circumference (cm)	31.5 (3.0)	27.6 (2.4)	<.001	9.00	229	
Δ Arm Circumferences (cm)	2.4 (1.1)	1.4 (0.9)	<.001	6.10	229	
Waist Circumference (cm)	78.6 (5.8)	70.0 (5.0)	<.001	9.99	229	
Hip Circumference (cm)	96.5 (6.8)	95.5 (7.2)	0.166	1.40	228	
WHR	0.8 (0.1)	0.7 (0.0)	<.001	9.89	228	
Sport Hours per Week	6.9 (3.3)	6.5 (4.1)	0.016	2.43	229	
^a^Actual Figure	3.8 (1.6)	5.2 (1.3)	<.001	−5.79	224	
^b^Ideal Figure	3.7 (0.9)	3.5 (1.0)	0.108	1.61	224	
^c^Body Dissatisfaction Score (BDS)	0.1 (1.4)	1.7 (1.3)	<.001	−7.63	224	
Weight Status^#^	%	%	<.001			19.2
Underweight	0.0	5.3				
Normal Weight	77.0	91.2				
Overweight	23.0	3.5				
Sport practice^#^	%	%				
Practice	77.6	57.9	0.004			8.3

^a^Subjects were shown nine silhouettes ordered from 1 to 9, from emaciation to obesity, and they selected the silhouette that they believed was most similar to their own (Actual Figure)

^b^Subjects were shown nine silhouettes ordered from 1 to 9, from emaciation to obesity, and they selected the silhouette that they most desired (Ideal Figure)

^c^BDS was calculated by subtracting the “ideal figure” from the “actual figure” score

^*^Comparisons were performed using Student’s t-test

^#^Comparisons were performed using chi-square

In females mean BMI and WHR values did not exceed the cut-off [32, 35]. The majority of females were normal weight (91.2%), while the prevalence of underweight and overweight was low (5.3% and 3.5% respectively) (Table 1). Only the 3.5% of subjects exceeded the WHR cut-off.

Considering weight status and fat distribution together, the females that present higher values than WHR cut-off belonged to a normal weight category.

A little more than half of females practiced sport, but the weekly sport hours were high (Table 1).

With regard to body image perception, females perceived themselves as slightly overweight (5.2) on an average, and chose an ideal figure in the normal weight category. As consequence, the BDS was positive, denoting dissatisfaction (1.7) (Table 1). In females actual and ideal figures, as well as BDS, were higher with increasing of weight status. Females in all of the weight categories tended to desire a thinner figure (Table 2), although actual figure was the only significant difference in females tested with ANOVA (df1, df2 = 53,2; F = 5.80; *p < 0.00*). Tukey’s post-hoc comparisons showed a statistical difference in actual figure between underweight and overweight subjects.

**Table 2:** Body image assessment by weight status

	Underweight Mean (SD)	Normalweight Mean (SD)	Overweight Mean (SD)	F (df1, df2)	*p* value
Females					
Actual Figure	3.0 (0.0)	5.2 (1.2)	6.5 (2.1)	5.80 (53,2)	0.005
Ideal Figure	2.7 (0.6)	3.5 (1.0)	4.5 (0.7)	2.15 (53,2)	0.126
BDS	0.3 (0.6)	1.8 (1.3)	2.0 (1.4)	1.94 (53,2)	0.154
Males					
Actual Figure	-	3.3 (1.4)	5.4 (1.1)	68.98 (168, 1)	<.001
Ideal Figure	-	3.6 (0.9)	4.1 (1.0)	7.42 (168, 1)	0.007
BDS	-	−0.3 (1.3)	1.3 (1.1)	49.31 (168, 1)	<.001

Statistical analyses were performed using ANOVA and Tukey’s post hoc test

*df* degree of freedom. *F* F values

Considering the difference in actual figures, ideal figures, and BDS between females who practice or not sport, a significant difference emerged for actual figure (df = 54, *T* = 2147, *p* = 0.036). Even if the BMI of two groups did not differ (sport = 21.7 vs. no sport = 21.5; df = 55, F = 1.07, *p* = 0.877), females that were not practicing sport perceived themselves larger (5.6 vs. 4.8). Males presented mean BMI and WHR values below the cut-off [32, 35]. As regards weight status, the majority of the sample was of normal weight (77.0%), only one subject was obese and the rest of the sample was overweight. The one subject in the obese category was grouped under the overweight category for further analyses. (Table 1). The frequency of subjects exceeding the WHR cut-off was 5.7%.

Considering weight status and fat distribution together, the males that presented higher values than WHR cut-off were normal weight (70%) or overweight (30%). 77.6% of males participated in sport and time spent participating in sport was an average of 6.9 h for week (Table 1).

As regards body image perception, males identified themselves as normal weight (3.8) and perceived themselves (ideal figure) in the same category (3.7); as consequence, the BDS was low (0.1). Analogously to females, actual and ideal figures, as well as BDS, were higher with increasing weight status. In males the differences between actual and ideal figures and BDS were always significant (Table 2), as the normal weight males desired a larger figure whereas the overweight males desired a thinner figure.

Considering fat distribution, no significant differences were noticed in the choice of actual and ideal figures and BDS between subjects of the same weight status category but exceeding or not cut-off for WHR. No statistical differences in actual figures, ideal figures, and BDS are detected between males who are engaged or not in sport (df = 168, *p* > 0.05).

Males did not present a correct perception of their weight status (chi-square = 49.60, *p* < 0.001) and they tended to categorize themselves in lower weight categories than those they actually belonged to (Table 3). Females misperceived their weight status as well (chi-square = 19.17, *p* < 0.001) (Table 3).

**Table 3:** Perceived body images of participants by their actual body weight status

	Weight status categories					
	Males %			Females %		
	Underweight	Normalweight	Overweight	Underweight	Normalweight	Overweight
Perceived Body Image:						
Underweight	-	60.3	0.0	100.0	9.8	0.0
Normalweight	-	32.1	57.9	0.0	43.1	50.0
Overweight	-	6.9	42.1	0.0	45.1	0.0
Obese	-	0.8	2.6	0.0	2.0	50.0

To get a more accurate assessment of the relationship between body image perception and anthropometric characteristics we performed a correlation analysis considering, in addition to BMI, also muscularity and fat distribution (Table 4). A significant correlation emerged only with BMI: in particular in males actual figure, ideal figure and BDS were significantly correlated with BMI, while in females only actual figure and BDS were correlated with BMI (Table 4).

**Table 4:** Correlation between actual figure, ideal figure, BDS and anthropometric indexes

	BMI	Δ Arm Circumference	WHR
Females			
Actual Figure	0.61	−0.08	−0.01
	*p* = <.001	*p* = 0.534	*p* = 0.958
Ideal Figure	0.20	0.01	0.08
	*p* = 0.132	*p* = 0.930	*p* = 0.537
BDS	0.49	−0.01	−0.07
	*p* = <.001	*p* = 0.465	*p* = 0.589
Males			
Actual Figure	0.65	−0.05	0.09
	*p* = <.001	*p* = 0.514	*p* = 0.226
Ideal Figure	0.24	0.03	0.01
	*p* = 0.002	*p* = 0.731	*p* = 0.873
BDS	0.58	−0.07	0.01
	*p* = <.001	*p* = 0.329	*p* = 0.203

To investigate the most informative predictors and their effects on actual figure, ideal figure and BDS, a hierarchical regression analysis was performed. According to the first model, being male was significantly associated with a lower self-perception score and BDS (Table 5).

**Table 5:** Hierarchical regression model to examine predictors for actual figure, ideal figure, and BDS

	Model 1				Model 2				Model 3			
Predictor Variables	β	T	CI	*p*	β	T	CI	*p*	β	T	CI	*p*
Actual Figure												
Gender (M)	−0.36	−5.79	(−0.92,-0.45)	<.001	−0.52	−8.27	(−1.23, −0.75)	<.001	−0.54	−8.44	(−1.27, −0.79)	<.001
BMI					0.63	12.47	(0.35, 0.49)	<.001	0.63	12.64	(0.36, 0.49)	<.001
Δ Arm Circumferences					−0.01	−0.13	(−0.16, 0.14)	0.895	−0.02	−0.39	(−0.18, 0.12)	0.698
WHR					−0.03	−0.47	(−3.78, 2.34)	0.641	−0.03	−0.47	(−3.76, 2.32)	0.641
Sport Hours per Week									0.01	0.21	(−0.05, 0.06)	0.831
Sport Practice (NO)									0.12	1.79	(−0.02, 0.48)	0.075
Gender/Sport Interaction									−0.13	−2.34	(−0.42, −0.04)	0.020
R^2^	0.13				0.49				0.51			
Adjusted R^2^	0.13				0.48				0.50			
*p*	<.001				<.001				<.001			
Ideal Figure												
Gender (M)	0.11	1.61	(3.44, 3.73)	0.108	0.02	0.25	(−0.16, 0.21)	0.800	0.00	−0.03	(−0.19, 0.19)	0.973
BMI					0.24	3.51	(0.04, 0.15)	<.001	0.25	3.62	(0.04, 0.15)	<.001
Δ Arm Circumferences					0.05	0.64	(−0.08, 0,16)	0.526	0.02	0.30	(−0.10, 0.14)	0.767
WHR					0.00	0.00	(−2.42, 2.42)	0.998	−0.01	−0.08	(−2.52, 2.31)	0.932
Sport Hours per Week									−0.14	−1.50	(−0.07, 0.01)	0.135
Sport Practice (NO)									−0.06	−0.63	(−0.27, 0.14)	0.528
Gender/Sport Interaction									−0.13	−1.74	(−0.29, 0.02)	0.083
R^2^	0.01				0.07				0.09			
Adjusted R^2^	0.01				0.05				0.06			
*p*	0.108				0.005				0.005			
BDS												
Gender (M)	−0.45	−7.63	(−1.01, −0.59)	<.001	−0.58	−8.83	(−1.24, −0.79)	<.001	−0.58	−8.73	(−1.26, −0.79)	<.001
BMI					0.53	10.11	(0.26, 0.39)	<.001	0.53	10.14	(0.26, 0.39)	<.001
Δ Arm Circumferences					−0.04	−0.66	(−0.19, 0.10)	0.509	−0.04	−0.65	(−0.19, 0.10)	0.518
WHR					−0.03	−0.49	(−3.67, 2.22)	0.628	−0.02	−0.41	(−3.54, 2.31)	0.679
Sport Hours per Week									0.10	1.46	(−0.01, 0.08)	0.147
Sport Practice (NO)									0.17	2.38	(0.05, 0.54)	0.018
Gender/Sport Interaction									−0.06	−1.00	(−0.28, 0.09)	0.318
R^2^	0.21				0.46				0.48			
Adjusted R^2^	0.20				0.45				0.46			
*p*	<.001				<.001				<.001			

Adding anthropometric characteristics to the model led to a significant increase of R2 in the second model. In addition to gender, higher BMI was significantly associated with higher actual figure values and body image dissatisfaction. BMI was the only significant predictor of ideal figure. When the third model is considered, including sport practice and sport hours per week, in addition to gender and BMI, the interaction between gender and sport practice proved to be a significant predictor of actual figure values. BMI remains the only significant predictor for ideal figure status. With regards to BDS, increasing BMI and lack of sport practice were significantly associated with higher body image dissatisfaction, whereas male gender, in general, was associated with a lower BDS.

## Discussion

Males in the present study had higher BMI values, a more centripetal fat distribution, and greater arm muscularity. The higher BMI value in males were in accordance with the results of other authors and therefore confirm the data from literature and does not point out any trend toward a reduction of BMI values in our sample [11, 36–38]. Some Authors reported an increasing incidence of eating disorders in young men in recent years and an increased social pressure on men to have low body fat and high muscularity [28, 39]. The findings of the present study indicated a high proportion of normal weight subjects among the sample and a prevalence of overweight, as reported in accordance other studies regarding Western and Southern European males, while lower for females [40].

Also regarding body image perception males confirm their tendency to perceive themselves slender. This observation was further confirmed by the reduced proportion of males who had a correct self-perception of their weight status, as they tended to underestimate their body size. This underestimation may be due to a denial of their own nutritional status [28, 41]. This misperception may impede efforts to prevent chronic degenerative diseases and other problems associated with excess weight in people with severe weight disorders. In contrast, normal weight and overweight women tended to overestimate their body size. Although both sexes prefer ideals of thinness, this tendency was more marked in females. Our study confirms the difference in body image self-perception between the two sexes, indicating that men may be paying less attention to their image [10, 41, 42]. Males who want to increase muscle mass could also desire a heavier body weight, since muscle tissue weighs more than adipose tissue [43].

In women, overestimation of body size may reflect environmental factors associated with the development of eating disorders [41, 44, 45]. Social expectations and stereotypes attached to gender in relation to body image may substantially influence weight perception and body image dissatisfaction. This may be associated with the conditioning factors in society that contribute to the stresses of modern life.

The desire for underweight and normal-weight women to have a more slender appearance may be instilled by mass media that promote pathologically underweight beauty models. The ideal body has undergone a substantial, as advertising and media outlets have propagated the representation of attractiveness as a thin, fit body shape for females, and a lean, muscular physique for males [5, 18, 42]. These beauty models spread through the media and are often influencing the behavior and food habits of children and adults [46, 47]. However, current standards of beauty are pervasive and unattainable for many individuals, and the pressure to achieve the elusive ideal body often leads to a diminished body image, increased eating disorders, and unsuccessful attempts at weight control [43]. Body prototypes for both men and women are becoming more restrictive and the media’s representation of males and females is changing [12, 13].

Weight status influenced actual and ideal figures as well as body dissatisfaction in our study, as they all rose with increasing weight status, in both genders. This trend was noticeable in females, as they showed a high prevalence of dissatisfaction with body weight and ideal body image. Considering sport practice, despite the BMI being similar, women who practiced sports perceived themselves slimmer than those who did not participate in sport.

In the present study men seemed not to still joining their female peers in the experience of body dissatisfaction, in contrast to Smith et al. [18]. Muscularity-oriented body image concern is becoming an increasing issue, but, contrary to expectations, the correlation between actual and ideal figures and BDS and muscularity in this study were not significant. The correlation between actual and ideal figures and BDS and fat distribution were also not significant. At this point it should be noted that the frequencies of subjects of the present study that exceeded the WHR cut-off were low in both sexes (5.7% in males and 3.5% in females), and this could have influenced the results.

On the other hand, the correlations with BMI were significant. To better understand the most informative predictors and their effects on actual figure, ideal figure and BDS, multiple regression models were carried out. These found that female gender was significantly associated with more overweight self-perception scores, thinner body ideal and consequently, higher body dissatisfaction.

Higher BMI was also significantly associated with a more overweight actual self-perception and increased body dissatisfaction. Subjects who did not involve in any sport activities presented significantly higher body dissatisfaction than those who participated in sports. A significantly greater proportion of females (almost twice as much as males) did not perform sport activities. The interaction between gender and sport activity significantly influenced the perceived actual figure status: being female and inactive increased actual figure perception. This was further confirmed by the statistically significant difference (*p < 0.05*) between active and inactive girls in terms of the actual figure perception.

In our study more active subjects (especially females) were more satisfied with their body image than their less active counterparts. This is in general agreement with other studies examining these associations in youth [21]. Our initial hypothesis that muscularity (based on the difference between the relaxed and contracted arm circumferences), distribution of body fat (as assessed by WHR), and the hours of sport activity may influence the perception of body image, was not confirmed by the results of our regression analyses. However, from the same analyses, sex, BMI and sport practice seemed to be the major correlates of body image perception and dissatisfaction.

An important strength of this study is that specialized personnel directly measured the sample of students, taking in account not only height and weight but also measures related to muscularity and fat distribution.

Limitations of this study must also be acknowledged. Due to the cross-sectional design used, although the model suggested possible directional relationships amongst variables, it is not possible to confirm causal pathways and further prospective research is needed in order to do this. The body silhouettes used to calculate body dissatisfaction differed only with respect to body fat, not muscularity, which makes the connections of body dissatisfaction with other variables in the study problematic, particularly for men.

In addition, a complete objective evaluation of physical activity was not carried out as the use of accelerometers was impracticable in this study.

## Conclusion

Overall, the present study highlighted a positive relationship between sport practice and body image perception, which underscores the importance of sport in improving self-consciousness, well-being, and health. Encouraging sport practice may improve sense of efficacy, feelings of self-determination, personal acceptance, and self-acceptance, and pursuing these positive effects may motivate individuals to initiate and maintain regular exercise routines. Dedicated long-term health education programs for students should focus on changing perceptions of body size and weight, emphasizing the importance of healthy lifestyle that includes sport practice, and popularizing a realistic and healthy body shape to debunk mass-media influences, especially among females.
